# Maturity-related acute effects of resistance exercise on muscle architecture and tissue organization of the resting and maximally isometrically contracted gastrocnemius medialis in girls

**DOI:** 10.3389/fspor.2026.1734509

**Published:** 2026-02-10

**Authors:** Melanie Lesinski, Luisa Walther, Gregory Bashford, Michael Cassel

**Affiliations:** 1 Division of Training and Movement Sciences, Research Focus Cognition Sciences, University of Potsdam, Potsdam, Germany; 2 Department of Biological Systems Engineering, University of Nebraska, Lincoln, NE, United States; 3 Department of Sports Medicine, University Outpatient Clinic, University of Potsdam, Potsdam, Germany

**Keywords:** isokinetic calf rises, isometric maximum voluntary contractions, maturity, post-pubertal, pre-pubertal, pubertal, spatial frequency analysis

## Abstract

**Objectives:**

This study aimed to examine maturity-related differences in the acute response of resistance exercise on the muscle architecture and tissue organization of the gastrocnemius medialis (GM).

**Method:**

Thirty-three healthy, physically active girls (11 pre-pubertal, 10 pubertal, 12 post-pubertal) conducted isometric maximum voluntary contractions (iMVC) before (pre), immediately after (post), and 15 min after (post_15) a resistance exercise (i.e., single leg isokinetic calf raises). Ultrasound scans were captured at pre, post, and post_15 during iMVCs as well as during rest. Muscle architecture measurements included muscle thickness (MT), superior and inferior pennation angles (PA), and fascicle length (FL). Spatial frequency analysis (SFA: peak spatial frequency radius [PSFR], peak −6 dB width [P6], quality factor [Q6], normalized peak value of amplitude spectrum [Amax], power within peak [PWP], peak power percent [PPP]) assessed muscle tissue organization.

**Results:**

Main effect of time reveals a significant acute increase in MT, Amax, PWP, and PPP from pre- to post-exercise, alongside a decrease in PSFR. However, analysis indicates no significant interaction between time and maturity group (*p* > 0.05). Concerning the main effects of the maturity group, the analysis shows significant differences: post-pubertal girls exhibit thicker MT, greater superior/inferior PA, and higher P6, but lower Q6, Amax, and PWP compared to pre-pubertal girls.

**Conclusions:**

The acute response suggests muscle swelling with increased fiber spacing and additional scattering material within the muscle, with no variation among girls of different maturity levels. Differences in muscle architecture and tissue organization between maturity groups highlight the role of biological maturation in muscular system.

## 1 Introduction

Maximum force is a fundamental component of sports performance, as it underpins powerful and explosive movements such as sprinting, jumping, throwing, and lifting (1). Maximum force is influenced by both morphological and neural factors (2). Research utilizing *in vivo* imaging techniques, such as ultrasound or magnetic resonance imaging, has provided clearer insights, revealing that muscle architecture characteristics such as muscle thickness (MT), pennation angle (PA), and fascicle length (FL) also impact force production capacity (3–5). Since these architectural parameters are closely linked to force-generating capacity, it is of particular interest to understand not only their role in determining maximal force but also how they adapt to different training stimuli. Changes in muscle architecture through resistance training have been widely studied. While the long-term effects of resistance training on muscle architecture have received considerable attention (6–8), the acute responses in muscle architecture following hypertrophy-focused training remain less explored. Some studies have highlighted acute muscle swelling in adults following resistance exercises such as exhaustive leg press (9), stretch-shortening cycle exercises (10), and isokinetic knee extensions (11). These changes are attributed to shifts in intra- and extracellular water balance within the muscle (9). In this context, increases in muscle thickness (MT) and pennation angle (PA) as well as a shortening of fascicle length (FL) have been observed due to acute muscle swelling (9, 11).

Beyond muscle architecture, muscle tissue organization at the micro-morphological level (1-50 µm) can now be examined using spatial frequency analysis (SFA). This quantitative ultrasound-based technique evaluates anisotropic speckle patterns in B-mode images via Fast Fourier Transformation (FFT) (11), generationg parameters such as: peak spatial frequency radius (PSFR), peak −6 dB width (P6), quality factor (Q6), normalized peak value of amplitude spectrum (Amax), power within peak (PWP), and peak power percent (PPP) that may reflect fiber alignment, packing density, or fiber density per unit volume (12). Lesinski et al. (12) reported transient alterations (i.e., normalization within 15 min) in these parameters following hypertrophy-focused resistance training during rest in adults, indicating short-lived structural changes without lasting muscle damage. This may result from increased fluid content (i.e., blood and/or water inflow) without reduced fiber organization, representing a normal physiological reaction (12).

While it is widely accepted that muscle architecture characteristics develop naturally throughout childhood and adolescence, research on the specific effects of resistance training on muscle architecture and tissue organization has primarily focused on adult populations. However, youth athletes have different training capacities, adherence, physical demands, physical conditions, muscel size, musle architecture, hormonal milieu, recovery capacity, and injury risks compared to adults (13–15), so the generalizability of previous research to children and youth is uncertain. Understanding these differences is important for training prescription, as maturity status may determine how muscle tissue acutely adapts and thus how load, volume, and recovery should be managed. It is also relevant for injury prevention, since mismatches between biological readiness and training stimuli can increase the risk of overload in less mature athletes. Finally, insights into maturity-specific responses may help explain how short-term adaptations translate into long-term hypertrophy and performance gains.

To date, however, no study has systematically investigated whether and how biological maturity stage modulates acute changes in muscle architecture and tissue organization following hypertrophy-oriented resistance exercise. Adressing this gap is essential for moving toward evidence-based, maturity-sensitive training concepts. This study aims to elucidate the acute changes, shedding light on the complex interplay between resistance exercise, acute muscle architecture and tissue organization adaptations as well as biological maturation. By combining conventional ultrasound with SFA, it provides novel insights into how maturation shapes acute muscular responses—knowledge that can serve as a foundation for developing maturity-specific training protocols aimed at enhancing performance while minimizing injury risks.

## 2 Methods and materials

### 2.1 Participants

Thirty-three healthy, physically active girls (aged 13 ± 4 years; height 152 ± 15 cm; weight 45 ± 16 kg; engaging in physical activity for 3.5 ± 2.4 h per week) volunteered to participate in the study. All characteristics of the participants, categorized by maturity group, are presented in Table 1. An *a priori* power analysis assuming a Type I error rate of 0.05 and a Type II error rate of 0.20 (i.e., 80% statistical power), conducted in accordance with Piponnier et al. (16), indicated that a sample size of 33 participants is sufficient to detect statistically significant maturity-related interaction effects.

**Table 1:** Characteristics of the participants.

Participant characteristics	Pre-pubertal (*n* = 11)	Pubertal (*n* = 10)	Post-pubertal (*n* = 12)	Mean (*N* = 33)
Age (years)	9.2 ± 1.2	12.3. ± 1.1	17.1 ± 2.2	13.0 ± 3.7
Standing height (cm)	134.4 ± 4.8	152.7 ± 8.3	166.4 ± 6.6	151.6 ± 15.0
Body mass (kg)	29.5 ± 5.4	41.4 ± 7.4	63.0 ± 9.7	45.3 ± 16.3
Maturity offset (years)	−2.5 ± 0.8	0.2 ± 1.0	3.8 ± 1.3	0.6 ± 2.8

To determine participants’ maturity status, we used the equation developed by Mirwald et al. (17), a non-invasive method for assessing biological maturity. Participants were divided into three maturity groups: pre-pubertal (>1.5 years before calculated maturity offset), pubertal (within ±1.5 years of calculated maturity offset), and post-pubertal (>1.5 years after calculated maturity offset).

To qualify as physically active, participants needed to engage in organized sports training outside of physical education at least once a week. Participants confirmed that they had not experienced any recent orthopedic conditions of the lower extremities or respiratory diseases within six months prior to the study's commencement. For all participants under the age of 18, parental consent was obtained.

All procedures adhered to the principles outlined in the Declaration of Helsinki and were approved by the local ethics committee (27/2022). Prior to participation, each subject received detailed information about the study's purpose, procedures, and potential risks. If subjects were minors, parents or guardians were also provided with this information. Written informed consent was obtained from both the subjects and their parents or guardians.

### 2.2 Measurement procedure

Testing procedures were conducted in a single testing session. All participants were tested individually. At the beginning of the testing session anthropometric data (i.e., standing and sitting body height, body mass), age, and physical activity habits were collected. Following this, a warm-up comprising 2 sets of 10 submaximal isokinetic plantar flexions (40° s^−1^, range of motion −20 to 35°) was conducted in an isokinetic device (Isomed2000, D&R Ferstl GmbH, Hemau, Germany). The main test session focused on hypertrophy-oriented resistance exercises, with participants performing 2 maximal isometric voluntary contractions (iMVCs) before (pre), immediately after (post), and 15 min after (post_15) the resistance exercises. Ultrasound scans were taken at pre, post, and post_15 during periods of rest and iMVCs. During rest, three ultrasound images were captured.

The resistance exercises were designed to promote muscle hypertrophy, with participants completing 4 sets of 10 repetitions of maximal isokinetic plantar extensions at 40° s^−1^. Rest intervals between sets lasted 90 s. After the resistance exercises, participants were instructed to relax for 15 min while maintaining a supine position.

### 2.3 Measurement of the isometric maximal voluntary plantar flexion

We evaluated iMVC of plantar flexion using an isokinetic dynamometer (Isomed2000, D&R Ferstl GmbH, Hemau, Germany). Participants underwent two measurements of iMVC for plantar flexion during pre and post tests. Each iMVC lasted for 5 s, with a 90-s rest between attempts. These measurements were conducted on the right lower leg of each participant. During the test, participants lay prone on the isokinetic device, with their knee fully extended to minimize movement at the ankle and knee joints. Their foot was securely held in place by a heel cup attached to a footplate, using toe and ankle straps, with the ankle positioned at 10° plantarflexion. Participants were instructed to exert maximum effort during each trial, with verbal encouragement provided by the investigators. The trial with the highest isometric torque from pre- and post-tests was chosen for further analysis.

### 2.4 Measurement of the muscle architecture and tissue organization

We conducted longitudinal ultrasound scans of the right GM muscle belly using a 7.5-MHz continuous linear ultrasound array (with a range of 4–13 MHz) on a Vivid q ultrasound system from GE Healthcare (Tirat Carmel, Israel). The ultrasound settings remained consistent throughout, with a frequency set at 11 MHz, depth at 4.5 cm, gain at 38%, dynamic range at 102, and focal points set at 1.2 cm and 2.5 cm for the GM muscle. These parameters were standardized for all image and video acquisitions. To stabilize the ultrasound probe, we used a custom-made foam holder and elastic Velcro straps (18). Acoustic coupling gel was applied between the transducer surface and the skin, with minimal pressure to avoid muscle compression. The measurement location of the GM muscle was predefined as one-third of the distance between the popliteal crease (tendon of the musculus semitendinosus) and the medial malleolus (18). To ensure precise identification in the ultrasound images and videos, we placed a thin strip of echo-absorptive tape 1.5 cm proximal to the marked spot (18, 19).

During pre- and post-tests, three ultrasound images in a resting state and two ultrasound videos capturing the contracted state during iMVC were obtained, maintaining a consistent prone position with fixed knee and ankle joint angles. These ultrasound videos were synchronized with iMVC force recordings. For analysis of the contracted state during iMVCs, a still image was extracted from the trial exhibiting the highest force in the iMVC force data.

Following each measurement, ultrasound images were saved and transferred to a computer for further analysis. We used ImageJ software (Version 1.53s, National Institutes of Health, Bethesda, MD) to assess skeletal muscle architecture, including MT, superior and inferior PA, and FL. MT was measured at a predetermined position 1.5 cm distal from the marking tape by measuring the distance between the upper and lower aponeuroses. Additionally, MT was measured at the most proximal and distal parts of the GM to determine aponeuroses alignment. Baudry et al. (20) proposed equations for FL calculation based on aponeuroses alignment (parallel or non-parallel) to account for cases where the fascicle extended beyond the ultrasound field of view.

Tissue micro-morphpological organization was assessed by importing all ultrasound images into MATLAB software (R2016a, MathWorks, USA) to perform SFA, following the methodology outlined by Bashford et al. (18, 19, 21). SFA is a quantitative ultrasound method that examines the anisotropic B-mode speckle pattern produced by tissue in the spatial frequency domain. In essence, this technique involves manually delineating a polygonal ROI on an image, within which smaller sub-regions referred to as “kernels” are examined in the spatial frequency domain. For each kernel within the ROI, parameters from the FFT-derived spatial frequency spectral estimate are extracted. The ROI was selected in a standardized manner by measuring a 1 cm wide rectangular area of the GM muscle, spanning from the upper to the lower aponeuroses at the muscle belly position (1 to 2 cm distal from the marking tape) (12, 19). Six spatial frequency parameters were calculated, including peak spatial frequency radius (PSFR), peak −6 dB width (P6), quality factor (Q6), normalized peak value of amplitude spectrum (Amax), power within peak (PWP), and peak power percent (PPP) (Table 2). Lesinski et al. (19) presented the mathematical description and physiological correlates of these six SFA parameters.

**Table 2:** Physiological correlate of spatial frequency parameters [based on Lesinski et al. (19)].

Parameter	Physiological correlates
peak spatial frequency radius (PSFR)	Dominant spacing of fascicles/fibers. Higher value primarily indicates a tighter packing of fascicles/fibers.
peak −6 dB width (P6)	Disorganization of fascicles/fibers. Higher value primarily indicates more disorganization of fascicles/fibers.
quality factor (Q6)	A normalization factor to facilitate comparision of fascicle/fiber spacing to disorganization. Higher value primarily indicates less disorganization relative to fascicle/fiber spacing.
normalized peak value of amplitude spectrum (Amax)	The strength of the most dominant fascicle/fiber spacing. Higher value primarily indicates higher fascicle/fiber density or perhaps lower fluid content.
power within peak (PWP)	The strength of a band of dominant fascicle/fiber spacings. Likely highly correlated to Amax. Higher value primarily indicates more fascicles/fibers that are organized compared to all scatterers.
peak power percent (PPP)	The power of organized fascicles/fibers compared to all (organized + disorganized) fibers. Higher value primarily indicates more tissue in alignment compared to other tissue in the sample volume.

Peak spatial frequency radius [PSFR], peak −6 dB width [Q6 = PSFR/P6], normalized peak value of amplitude spectrum [Amax], power within peak [PWP], peak power percent [PPP.

Image analysis of muscle architecture and tissue organization was performed blinded. The reliability of the muscle architecture and tissue organization measurement methodology was confirmed in Lesinski et al. (18), who reported intraclass correlation coefficients ranging from 0.62 to 0.996 for the different outcome parameters when repeatedly measuring the medial gastrocnemius muscle.

First, it is important to emphasize that we have a solid understanding of the *image-based* meaning of each SFA parameter. However, their physiological correlates remain largely theoretical. Some are more well-established (e.g., PSFR), while others (e.g., PWP and PPP) are less clearly understood. Additionally, there is likely to be some overlap in the physiological interpretation of these parameters.

### 2.5 Statistical analysis

Normal distribution was assessed using the Shapiro–Wilk test. A 3 (maturity group: prepubertal, pubertal, postpubertal) × 3 (time: pre, post, post_15) analysis of variance (ANOVA) with repeated measures on time was conducted to investigate the impact of maturity and resistance exercise on muscle architecture and tissue organization during rest and iMVC. *Post-hoc* tests with Bonferroni-adjusted *α* were carried out to identify statistically significant comparisons. Additionally, effect sizes were determined using Cohen's *d*, calculated from eta-squared, and classified as small (*d* < 0.5), medium (0.5 ≤ *d* < 0.8), or large (*d* ≥ 0.8), following Cohen's (1988) guidelines. All statistical analyses were conducted using Statistical Package for the Social Sciences (SPSS) version 29.0, with the significance level set at *p* < 0.05.

## 3 Results

### 3.1 Main effects of time on muscle architecture and tissue organization

The main effects of time (i.e., pre, post, post_15) are shown in Table 3.

**Table 3:** Main effects of time and group as well as interaction effects of time × mautrity group during rest and isometric maximal voluntary contraction (iMVC).

Effects		During rest						During iMVC					
		Time		Maturity group		Time × maturity group		Time		Maturity group		Time × maturity group	
Parameter		*p*-value	Cohens *d*	*p*-value	Cohens *d*	*p*-value	Cohens *d*	*p*-value	Cohens *d*	*p*-value	Cohens *d*	*p*-value	Cohens *d*
Muscle architecture	muscle thicknessT	0.000	2.49	0.001	1.55	0.569	0.47	0.308	0.40	0.000	1.63	0.353	0.55
	superior pennation angle	0.604	0.26	0.007	1.26	0.246	0.61	0.042	0.67	0.008	1.23	0.390	0.53
	Inferior pennation angle	0.061	0.63	0.002	1.45	0.424	0.52	0.027	0.71	0.000	1.74	0.253	0.61
	fascicle length	0.458	0.33	0.318	0.56	0.538	0.45	0.421	0.34	0.414	0.49	0.610	0.43
Tissue organization	PSFR	0.006	0.86	0.091	0.83	0.174	0.62	0.102	0.56	0.208	0.66	0.204	0.64
	P6	0.363	0.37	0.175	0.70	0.111	0.67	0.044	0.66	0.007	1.25	0.188	0.65
	Q6	0.069	0.50	0.013	1.16	0.370	0.54	0.089	0.58	0.231	0.64	0.089	0.75
	Amax	0.000	1.09	0.177	0.70	0.109	0.69	0.181	0.48	0.012	1.18	0.455	0.50
	PWP	0.000	1.12	0.190	0.68	0.262	0.59	0.370	0.37	0.018	1.11	0.429	0.51
	PPP	0.003	0.94	0.148	0.74	0.198	0.62	0.074	0.60	0.041	0.97	0.260	0.60

**Figure 1:**
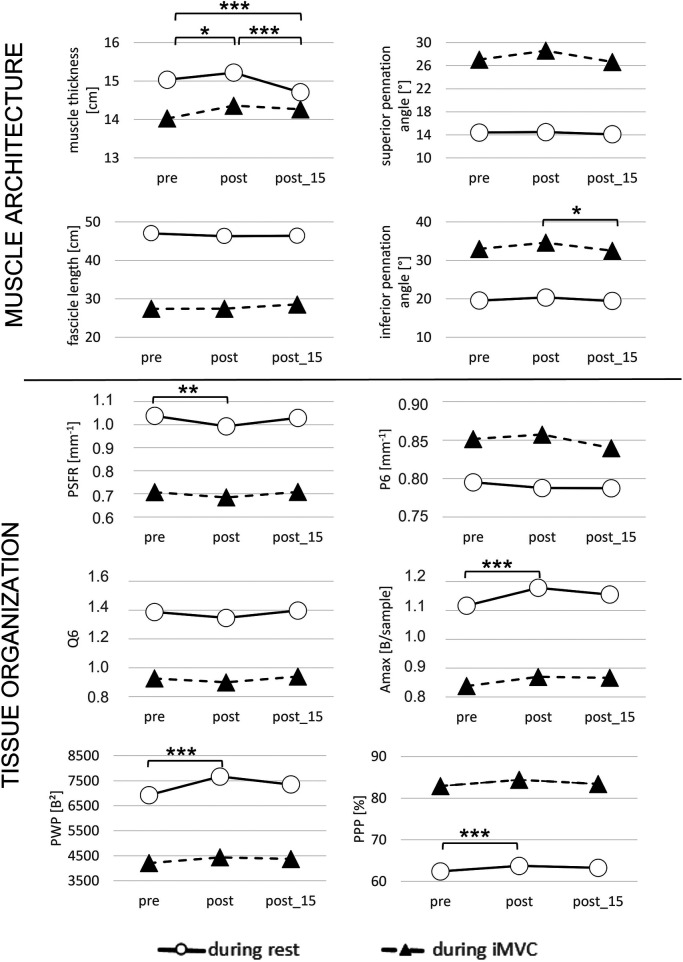
Main effects of time: muscle architecture and spatial frequency analysis parameter pre, post, and post_15 during rest and isometric maximal voluntary contraction (iMVC). Legend: * = *p* < 0.05; ** = *p* < 0.01; *** = *p* < 0.001.

Regarding muscle architecture, only MT changed significantly at rest during time. *Post-hoc* analysis revealed that MT significantly increased from pre to post (+1.3%; Figure 1) and significantly decreased from pre/post to post_15 (−2.1% to −3.4%; Figure 1). During iMVC, superior and inferior PA changed significantly over time. *Post hoc* analysis revealed that inferior PA significantly decreased from post to post_15 (−4.4%; Figure 1).

Regarding tissue organization, the analysis showed that PSFR, Amax, PWP, and PPP significantly changed over time. *Post-hoc* analysis indicated that PSFR significantly decreased from pre to post (−4.8%; Figure 1), while Amax (+5.4%), PWP (+10.7%), and PPP (+2.1%) significantly increased from pre to post at rest (Figure 1). During iMVC, *post-hoc* test revealed no significant differences in any tissue organization parameter over time.

### 3.2 Main effects of maturity group on muscle architecture and tissue organization

The main effects of maturity group (i.e., pre-pubertal, pubertal, post-pubertal) are shown in Table 3.

**Figure 2:**
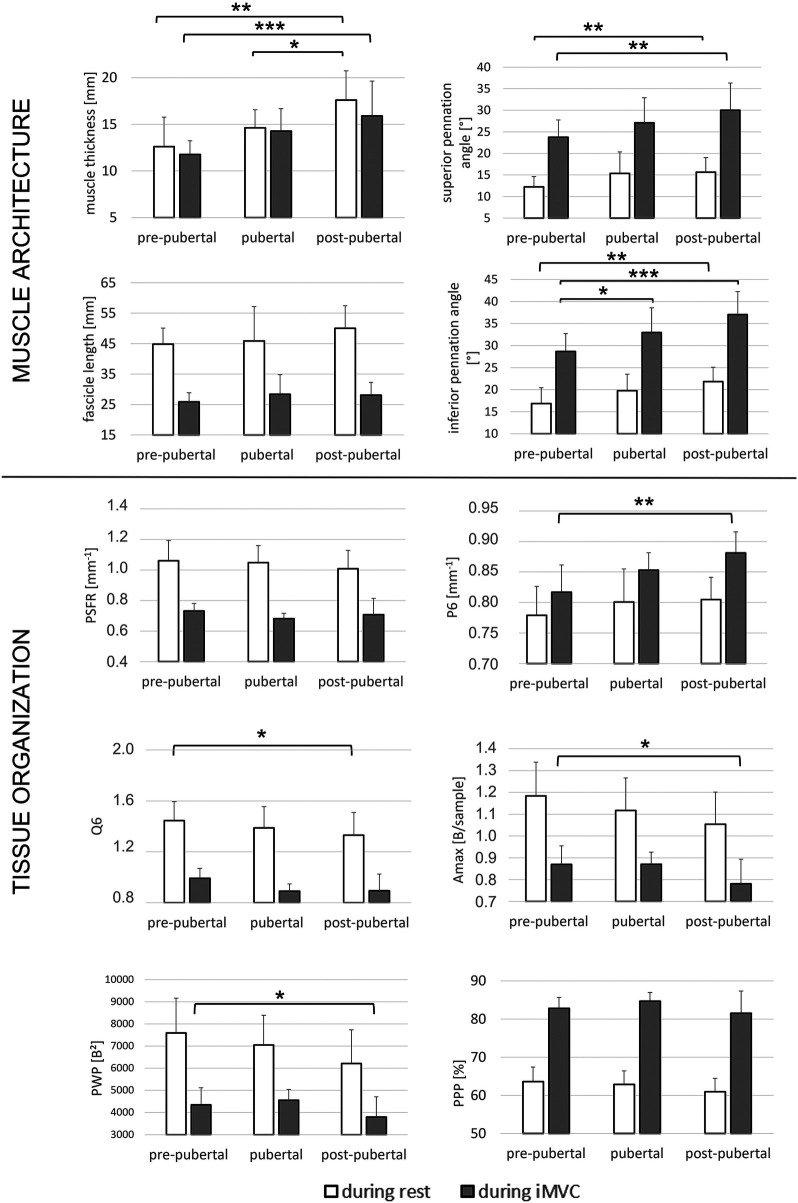
Effects of maturation: mean and SD of muscle architecture and spatial frequency analysis parameters of pre-pubertal, pubertal and post-pubertal during rest and isometric maximal voluntary contraction (iMVC). Legend: * = *p* < 0.05; ** = *p* < 0.01; *** = *p* < 0.001.

Regarding muscle architecture, the analysis revealed significant increases in MT (+39.0-39.7%), as well as in superior (+28.8-33.5%) and inferior PA (+26.3-32.6%), with advancing maturation, both at rest and during iMVC (Figure 2).

Regarding tissue organization, the analysis showed that at rest, Q6 was significantly higher in pre- compared to post-pubertal girls (+11.6%). During iMVC, the analysis demonstrated that P6 was significantly lower in pre-pubertal compared to post-pubertal girls (−6.1%), while Amax (+13.2%) and PWP (+17.3%) were significantly higher in pre-pubertal compared to post-pubertal girls (Figure 2).

### 3.3 Time × maturity group interaction effects on muscle architecture and tissue organization

There were no significant time  ×  maturity group effects on any of the muscle architecture and tissue organization parameters (Table 3).

## 4 Discussion

The purpose of this study was to investigate the maturity-related acute effects of hypertrophy-focused resistance exercise on muscle architecture and tissue organization at rest and during iMVC of the GM muscle in girls. The main effect of time (i.e., effects of resistance exercise) indicates muscle swelling, reflected by increased MT and additionally suggested by potentially greater spacing between muscle fibers and the possible presence of additional scattering material (i.e., fluid) within the muscle, as inferred from changes in specific SFA parameters. These acute changes in muscle architecture and tissue organization are particularly evident at rest. Specifically, from pre- to post-exercise, MT, Amax, PWP, and PPP increased, while PSFR decreased. Nevertheless, these general short-term effects of hypertrophy-focused resistance stimuli were consistent regardless of biological maturation (i.e., no time  ×  maturation group interaction effect). With respect to the main effects of maturity group, the analysis revealed significant differences in muscle architecture and tissue organization. Compared to pre-pubertal girls, post-pubertal girls showed thicker MT, greater superior and inferior PA, and lower values for Q6, Amax, and PWP, along with higher P6. Finaly, muscle architecture and tissue organization were significantly different in the resting state compared to during iMVC.

First, pronounced significant acute changes in muscle architecture and tissue organization were observed over time, particularly during rest. There was an increase in MT, Amax, PWP, and PPP from pre- to post-exercise, while PSFR significantly decreased. The swelling of the muscle (i.e., an increase in MT) may lead to greater spacing between fibers, which is likely indicated by a reduction in PSFR. The concurrent increases in Amax and PWP likely reflect the presence of additional scattering material (i.e., fluid) within the muscle tissue. It remains to be clarified whether a quantitative analysis of MT and, in particular, tissue organization using SFA can serve as a tool to monitor individual training load and thereby provide insights into the actual internal stress experienced. When comparing these results with those of Lesinski et al. (12), differences between healthy, trained girls and healthy, trained young adults (i.e., sports science students, both men and women, 25 ± 3 years) become evident. In adults, muscle architecture adaptation at rest were limited to an increase in MT, while during iMVC, both MT and FL increased significantly, accompanied by a reduction in superior PA (12). Thus, the architectural responses of adults and girls were not fully identical. With regard to tissue organization, Lesinski et al. (12) found altered parameters only during rest, including a significant drop in PSFR and Q6 from pre- to post-exercise. These changes were transient, as SFA parameters normalized within 15 min. Similarly, in girls, significant changes in tissue organization were observed only at rest and returned to baseline within the same time frame. However, in addition to a significant PSFR decrease—also observed in adults and may be indicative of muscle swelling—girls exhibited significant increases in Amax, PWP, and PPP, which were absent in adults. This may suggest that less scattering material (i.e., fluid) is recruited into the muscle of older individuals after exercise, whereas younger individuals may experience a greater influx of fluid into the muscle tissue during or immediately after exercise. Taken together, these findings suggest that children and adolescents display somewhat different acute responses to a hypertrophy-oriented resistance stimulus compared to adults. Several explanations are possible. Methodological differences between studies may have contributed, since adults performed single-leg calf raises whereas the girls performed isokinetic plantar flexion on an Isomed. Nevertheless, it appears more likely that the differences reflect physiological mechanisms, particularly variations in fatigue resistance and recovery capacity. For instance, while the acute increase in MT persisted for at least 15 min in adults, it had already subsided within the same period in girls, indicating faster recovery in younger participants. This distinct behavior can be attributed to developmental differences in intramuscular conditions and exercise responses (22). Children typically have a more favorable muscle fiber composition, with a higher proportion of fatigue-resistant type I fibers, as well as a more efficient metabolic profile—characterized by lower blood and muscle lactate accumulation and faster phosphocreatine resynthesis (22). Together, these factors enable a quicker recovery of muscle fibers in children and adolescents compared to adults.

Moreover, our results indicate that general muscle architecture and tissue organization differ significantly between maturity groups in girls. We found that MT is greater and both superior and inferior PA are larger in biologically more mature girls, regardless of whether tested at rest or during iMVC. This supports the assumption that muscle architecture develops progressively with growth and maturation (e.g., increases in MT and PA) (23, 24). Muscle growth during development is primarily driven by hypertrophy of existing fibers, as fiber number remains largely constant throughout life (25). Satellite cells contribute additional myonuclei, supporting protein synthesis and the addition of sarcomeres both in series (i.e., longitudinal muscle growth) and in parallel (i.e., hypertrophic muscle growth) (25, 26). These structural changes are further promoted by rising body mass, mechanical loading from daily activity and sport (27), and hormonal changes such as elevated testosterone during puberty (28). Examining tissue organization, we observe lower Q6, Amax, and PWP, as well as higher P6 in post-pubertal compared to pre-pubertal girls. This could suggest that the younger group may have more organized tissue (increased Q6, lower P6), which might be reflected by smaller fiber groups. Although not statistically significant, PSFR appears to decrease with biological age. The tighter fiber bands could also potentially explain the higher Amax and PWP. To date, no studies have examined muscle architecture and tissue organization in girls at different stages of biological maturity using sonographic analysis. Our findings provide preliminary insight into the influence of biological maturation on muscular development, and future examination of tissue organization may help explain injury susceptibility—for example, why muscle injuries are far less frequent in prepubertal children compared to (post-)pubertal individuals (29, 30).

Nevertheless, despite the observed differences in muscle architecture and tissue organization between prepubertal, pubertal, and postpubertal girls, they exhibited the same acute response to the applied hypertrophy-focused resistance stimulus (i.e., no significant time × maturity group interaction effects). The comparable acute responses across girls of different biological maturity may reflect either the absence of true differences in the acute reaction, as these responses are more global—such as fluid shifts and intramuscular perfusion that appear to be similar across age groups—or the limited sensitivity of SFA and ultrasound to detect subtle molecular or cellular changes, which could contribute to the observed similarity. In the short term, no differentiation within the studied cohort was possible, and it remains unclear whether for instance long-term training adaptations would reveal maturity-dependent differences. Examining tissue organization may provide valuable insight in this context. These aspects warrant further investigation.

Finally, the test results reveal significant differences in muscle architecture and tissue organization during rest and iMVC, despite maintaining constant knee and ankle angles. This is consistent with the findings of Lesinski et al. (12), who demonstrated that MT and FL are greater at rest than during iMVC, while superior and inferior PA are smaller at rest compared to iMVC. These findings suggest that isometric contraction shortens the contractile elements without altering the overall muscle length, resulting in a steeper angle of the contracted (shortened) muscle fibers. Regarding micromorphology, in accordance with Lesinski et al. (12), it was observed that PSFR, Q6, Amax, and PWP are significantly higher in the resting muscle compared to the isometrically contracted muscle, whereas P6 and PPP are significantly lower. This once again suggests that the transition from rest to iMVC may lead to contraction-specific changes at the micromorphological level. One possible explanation for these observations is that the contraction of muscle fibers could result in an apparent decrease in fiber alignment (i.e., as may be indicated by higher P6 and lower PSFR in the ultrasound image), an apparent increase in fluid density (i.e., as may be indicated by lower Amax), and an increase in the relative amount of tissue alignment compared to non-fiber structures (i.e., as may be indicated by higher PPP) (12).

One limitation of this study is the focus on the muscle belly. Resistance exercises can elicit different responses depending on whether the region near the tendon insertions (proximal, distal) or the mid-belly is examined. Due to the constraint of using a single ultrasound probe, we chose to examine the muscle belly. This decision was based on the reliability of muscle architecture and SFA parameters at the muscle belly demonstrated in two previous studies (18, 19). However, it should be noted that proximal or distal muscle regions may respond differently. Another potential limitation relates to participant characteristics. Anthropometric differences may have acted as potential confounders. Although several outcomes showed moderate effect sizes, no significant interaction effects were detected between maturity group and time. This makes it unclear whether the findings reflect true physiological similarity across maturity stages or are partly attributable to limited statistical power. While an *a priori* sample size calculation was performed for this exploratory study, it was based on limited prior data, as the reference study examined maturity-by-time interactions after acute resistance exercise but did not include specific ultrasound outcomes. This may have led to an underestimation of the sample size required to detect interaction effects for the ultrasound variables. Although the overall sample size is appropriate for an experimental study involving children, the small group sizes (*n* = 10–12 per maturity group) reduced sensitivity to detect interactions. Therefore, the absence of statistically significant interaction effects should be interpreted with caution, as some effects with non-negligible effect sizes might have reached significance in a larger sample. A further limitation concerns the estimation of biological age. The Mirwald calculation provides a practical, non-invasive estimate of biological age, but it has several limitations (31–33). Accuracy may be reduced in very early or late pubertal stages, in children with extreme body sizes or weights, and across different populations. Additionally, it does not capture hormonal, genetic, or other physiological factors influencing maturation, and its linear assumption of growth relative to maturity may not fully reflect the non-linear nature of pubertal development. Therefore, estimates should be interpreted as approximate rather than definitive measures of biological age. Finally, the absence of a familiarization session for the girls with the Isomed device constitutes another limitation. As this was a laboratory study, it was challenging to invite the minors to the lab twice due to logistical reasons. Nonetheless, the girls received thorough instructions, had a practice/warm-up phase with the device, and were trained and thus had good motor control.

## 5 Conclusion

In conclusion, this study demonstrated the acute responses of muscle architecture and tissue organization to hypertrophy-focused resistance exercise in pre-pubertal, pubertal, and post-pubertal girls, providing new insights at both macro- and micro-morphological levels. Across all maturity groups, the acute response was characterized by muscle swelling, including increased fiber spacing and fluid accumulation within the tissue. Nevertheless, hypertrophy-focused resistance stimuli elicited similar acute responses across girls at different stages of biological maturation (no interaction effect). Significant general differences in muscle architecture and tissue organization between maturity groups underscore the impact of biological maturation on the muscular system.

While the image-based interpretation of each SFA parameter is well understood, their physiological correlates remain largely theoretical, with some (e.g., PSFR) better established than others (e.g., PWP, PPP). Nevertheless, SFA allows for a detailed assessment of muscle micro-morphology through sonografic analysis, providing researchers and clinicians with valuable insights into individual intramuscular structure and adaptations. In the future, SFA analyses could offer substantial benefits in research by enabling, for example, the assessment of sex differences, the effects of different training methods, long-term training adaptations, and informing clinical approaches to injury susceptibility.

## Data Availability

The raw data supporting the conclusions of this article will be made available by the authors, without undue reservation.
